# Exploring the determinants of reinvestment decisions: Sense of personal responsibility, preferences, and loss framing

**DOI:** 10.3389/fpsyg.2022.1025181

**Published:** 2023-01-12

**Authors:** Johannes T. Doerflinger, Torsten Martiny-Huenger, Peter M. Gollwitzer

**Affiliations:** ^1^Department of Psychology, University of Konstanz, Konstanz, Germany; ^2^Department of Psychology, UiT The Arctic University of Norway, Tromsø, Norway; ^3^Department of Psychology, New York University, New York, NY, United States

**Keywords:** investment decisions, escalation of commitment, personal responsibility, framing, preferences, poker game, sunk cost

## Abstract

Two potentially costly errors are common in sequential investment decisions: sticking too long to a failing course of action (escalation of commitment), and abandoning a successful course of action prematurely. Past research has mostly focused on escalation of commitment, and identified three critical determinants: personal responsibility, preferences for prior decisions, and decision framing. We demonstrate in three studies using an incentivized poker inspired task that these determinants of escalation reliably lead decision makers to keep investing even when real money is on the line. We observed in Experiments 1, 2 and 3 that reinvestments were more likely when decision makers were personally responsible for prior decisions. This likelihood was also increased when the decision makers had indicated a preference for initial investments (Experiments 2 and 3), and when outcomes were framed in terms of losses as compared to gains (Experiment 3). Both types of decision errors – escalation of commitment and prematurely abandoning a course of action – could be traced to the same set of determinants. Being personally responsible for prior decisions, having a preference for the initial investment, and loss framing did increase escalation, whereas lacking personal responsibility, having no preference for the initial investment, and gain framing increased the likelihood of prematurely opting out. Finally, personal responsibility had a negative effect on decision quality, as decision-makers were still more likely to reinvest when they were personally responsible for prior decisions, than when prior decisions were assigned optimally by an algorithm (Experiments 2 and 3).

## 1. Introduction

When individuals repeatedly face the decision to further invest in or opt-out of a course of action, accurately using the available information is crucial to avoid two potential decision errors: first, abandoning the successful course of action too early and thus missing out on potential benefits, and second, persisting too long with a futile course of action (Drummond, 2014). The latter refers to one of the major branches of the sunk cost research (progress decisions; Roth et al., 2015) and is often labeled as escalation of commitment (EoC). The effect has been observed in a multitude of domains (e.g., personal, business, political, or gambling decisions; Sleesman et al., 2012), both on the individual and the group level (Sleesman et al., 2018). Research has identified factors enhancing EoC: among others personal responsibility for prior decisions (Staw, 1976), preferences for the initial decisions (Schulz-Hardt et al., 2009), and increased risk-seeking when dealing with losses (Soman, 2004).

Although there are experimental studies investigating EoC most of these relied on hypothetical scenarios (reviewed by Roth et al., 2015; Sleesman et al., 2018; see also Negrini et al., 2020), with some exceptions (e.g., Heath, 1995; Wong et al., 2006; Ronayne et al., 2021). More importantly, there are almost no studies investigating the responsibility/self-justification factor in experimental designs with real consequences. A study by Kirby and Davis (1998) is an exception. Kirby and Davis introduced a complex company setup in which participants solved anagrams using a specific strategy. In the escalation decision, participants could invest money into continuing with the (failing) strategy that they had previously chosen themselves or not. The results show an effect of responsibility; participants that made the first strategy decision invested more money into continuing with that strategy. There are some noteworthy details. The study involved a complex setup including an indirect relation between study performance and the real money to be gained, and the study setup relied on considerable false information given to participants. First, the real consequences were not directly contingent on the participants’ decision. Instead, the money that participants ended up with as profit in their “company” were exchanged into raffle tickets. Thus, making more money in the study’s game only increased the likelihood of winning the raffle. In addition, these supposedly real consequences were actually not implemented and all participants received the same amount of raffle tickets, a procedure that is not in line with the strict standards of behavioral economics research. Furthermore, the negative feedback that participants received regarding the initially chosen strategy was false information that was the same for all participants.

In sum, considering the prominence of the responsibility/self-justification factor in the literature on escalation of commitment and the limited experimental evidence for it, there is a need to validate this EoC determinant. Such validation does not only require study designs that focus on manipulating the critical factors but also on assessing actual task performance.

### 1.1. Determinants of escalation of commitment

A standard experimental task paradigm in EoC research (Staw, 1976) is confronting research participants with hypothetical investment scenarios, in which research participants are asked to take on the role of a CEO and then choose one of two investment alternatives. For example, participants are asked to select one of two projects in a company – the company could either develop consumer products or industrial products – in which they could hypothetically invest $8 million. Participants then receive either positive or negative feedback, meaning that the project they had invested in has done well or poorly. Following this feedback, participants are asked to allocate a given amount of money (e.g., $10 million) between the previously chosen and the non-chosen alternative. Reinvesting in the previously chosen option after negative feedback is regarded as a suboptimal strategy and labeled as EoC (e.g., Lipsey and Harbury, 1992). In such research, multiple psychological mechanisms and situational influences were found to affect EoC.

#### 1.1.1. Personal responsibility and preferences for the initial decision

One of the most prominently studied determinants for EoC is personal responsibility: If decision-makers are personally responsible for initial investments and experience that their chosen course of action is failing, they are driven to stick with it as a form of ego-defense. For example, in hypothetical scenarios, participants are either asked to make the first investment decision or are told that this decision had been made by their predecessor. A common finding is that participants who made the first investment decision will later invest more money than participants who did not make this decision, even if the course of action invested in is failing (Staw, 1976; Bobocel and Meyer, 1994).

Schulz-Hardt et al. (2009) argued that decision-makers persist with a chosen course of action not because of a responsibility bias but because they originally had a higher preference for the chosen rather than the non-chosen option. As choices are to some extent guided by preferences, there is a greater fit between the choices and preferences in conditions in which decision-makers make the decision themselves compared to conditions in which the decision-makers do not make the decision themselves. Tasks in which only personal responsibility is manipulated thus confound personal responsibility with preferences. The authors found that preferences for the initial decision fully mediated the effects of manipulated personal responsibility on subsequent hypothetical reinvestment decisions (Study 1) or on sticking to a chosen strategy of task performance (Study 2).

Besides Schulz-Hardt et al. (2009), very few studies directly measured preferences for the initial investment decisions in an EoC task. A meta-analytic review by (Sleesman et al., 2012) found a small positive main effect of preferences for the initial decision on EoC. The authors concluded that this preference effect was not strong enough to fully account for responsibility effects. They also categorized studies into those in which the decision-makers actually made the initial decision themselves versus those in which the initial decision was merely assigned to the participants. Sleesman et al. argued that studies requiring an actual initial decision should consist mostly of participants who prefer the chosen course of action, whereas studies in which the initial decision is assigned should consist of both participants with and without a high preference. Using this categorization of actually made versus adopting assigned decisions, the authors however found no difference in EoC. Whereas the authors conclude that this finding raises doubts regarding a preference-based explanation of EoC, it also raises questions regarding the responsibility explanation. Should one not expect to see more substantial personal responsibility effects in studies where participants are actually responsible instead of just being told that they are responsible even though they were not?

Taken together, evidence suggests that preferring the initially chosen option over the non-chosen option increases the likelihood of reinvesting in a chosen course of action. We expect that this will not just be the case in EoC situations – preference effects should also be a driver of continued investments, when reinvesting is the prudent course of action. However, it remains unclear whether preferences can fully account for personal responsibility effects. We therefore measured preferences and assessed whether there are personal responsibility effects beyond them.

#### 1.1.2. Framing

EoC has also been interpreted as a consequence of negative decision framing (Thaler, 1980; Whyte, 1986; Arkes, 1991; Soman, 2004); decision makers are assumed to construe their previous investments after failure feedback in terms of losses. Prospect theory (Kahneman and Tversky, 1979; Tversky and Kahneman, 1992) postulates that when people are dealing with gains, they are less sensitive to additional gains – high gain options with the risk for low (or no) gains are less attractive than risk-free moderate gain options. However, when people are dealing with losses, they are less sensitive to additional losses – no (or low) loss options with the risk for high losses are more attractive than risk-free moderate loss options. Typical EoC situations can be mapped onto the gain/loss framework described in prospect theory. When actors think about prior investments in terms of losses, additional (risky) investments may be perceived as an opportunity to avoid or recoup losses. Whether decision makers construe a situation in terms of either gains or losses can be manipulated by framing the outcomes accordingly (Tversky and Kahneman, 1981).

Experimental research on gain vs. loss framing and EoC is limited so far. Rutledge (Rutledge, 1995) examined EoC and framing effects in a modified investment task for small groups with a gain versus loss framing manipulation. Participants in their study were asked to assume the role of financial vice presidents of a fictitious company. They worked in groups of three and were asked to decide whether to make a reinvestment decision for a failing project. Personal responsibility was manipulated by telling participants that the initial investment decision was made because they had recommended it themselves or because of the recommendation of another team. Consequences were presented for half the participants in terms of savings and the other half in terms of losses. The author observed personal responsibility and framing effects on EoC; responsibility effects were more pronounced in the loss frame than in the gain frame condition. This finding is in line with the prospect theory account of EoC, which predicts that loss framing should increase escalation of commitment. However, Schoorman et al. (1994) manipulated the gain/loss framing in hypothetical investment scenarios and observed framing effects only when little (vs. much) context information was given.

In sum, typical EoC tasks put participants into a situation that prospect theory would refer to as loss framing. Prospect theory and studies on EoC converge on predicting that participants are likely to take risks beyond what might be considered reasonable from a probability perspective. However, experimental evidence regarding gain/loss framing and reinvestments is limited so far and based solely on using hypothetical decision scenarios. Also, the question remains whether moving Rutledge’s (1995) research conducted at the group level onto individual (non-hypothetical) decision-making results in comparable findings. If this is the case, then responsibility effects should be even more pronounced if outcomes are framed as losses. In any case, individuals should be more likely to invest in a loss than in a gain frame.

## 2. Present research

Prior research on EoC predominantly relied on hypothetical scenarios tasks in experimental studies, but the determinants are also supported by non-experimental studies examining investments on the organizational level [e.g., McNamara et al., 2002; Hsieh et al., 2015 review by Sleesman et al. (2018)]. We used a poker-game inspired computer task (VIP-Task; Doerflinger et al., 2017) that allowed us to conjointly manipulate the previously identified features (i.e., personal responsibility, loss/gain framing) and measure relevant variables for each decision (i.e., preferences) in a context with real consequences for participants.

Poker is a card game of chance and strategy, in which the players have to repeatedly decide whether to bet on their cards (i.e., invest further resources) or opt-out. Opting out means disregarding some still existing chances of winning and incurring a sure loss, but potentially avoiding throwing good money after bad. Between each bet, the chances of winning can change. Leaving strategic social interactions (e.g., bluffing) aside, poker players should only consider prospective gains and losses to arrive at the best outcome. Nonetheless, players regularly fall victim to EoC effects or miss out on good investments (Smith et al., 2009). The quality of poker decisions depends highly on the probability of success. The same is true for reinvestment decisions in many other real-life domains (e.g., in a business, there is the probability that competitors whom I did not yet know of enter the stage). Therefore, we see poker decisions as a suitable model environment for testing reinvestment decisions in realistic incentivized experiments.

The purpose of Experiment 1 is to demonstrate the personal responsibility effect on performing the VIP-Task. In Experiment 2, we tested whether personal responsibility effects still occur when initial preferences were controlled for; are personal responsibility effects independent of whether people prefer the initially chosen option or not? Experiment 3 was designed to test the effects of responsibility, preferences, and gain/loss framing of outcomes in parallel. The design of Experiments 2 and 3 allows evaluating the quality of those decisions where the participants chose to invest and those decisions where they chose to opt out based on the expected value of the decision. Experiments 2 and 3 include two benchmarks against which we compare participants’ reinvestment decisions: decisions when the prior investment was made by an algorithm either (1) randomly or (2) optimally in line with expected-value principles. We obtained approval from the university’s ethics committee for all of the studies reported in the present manuscript. We used the simr package for R (Green and MacLeod, 2016) to estimate the statistical power *via* simulations. The sample size and number of trials in all three experiments are sufficient to detect small within-participants effects (OR = 1.4) with a probability of *β*-1 > 0.95 at the *α* = 0.05 significance level in mixed effects logistic regressions with random effects for participants (Experiments 1, 2, and 3) and for trials (Experiment 1). Experiment 3 was preregistered, and the data for all three experiments and a pilot study for Experiment 3 are available at: https://osf.io/hdczr/?view_only=546aec80e6f7468685072d199a9d9821.

### 2.1. Experiment 1: Personal responsibility

Participants played multiple rounds of a poker inspired card game against a computer. To increase their payout, they had to repeatedly decide whether to keep investing into new cards or to quit a round. We tested responsibility effects on reinvestments by asking participants to make reinvestment decisions after having made prior investment decisions or having adopted prior investment decisions made by the computer.

#### 2.1.1. Method

##### 2.1.1.1. Participants and design

Fifty-one individuals (39 female) with a mean age of 23.0 (range 19 to 41, SD = 4.9) recruited at a German university participated. Personal responsibility was manipulated as a within-participants factor with two levels (personally responsible vs. assigned). A prior investment factor resulted from the repeated investment in a round where a given decision was preceded by one to four prior investments. Losing probability was calculated as a quasi-experimental predictor for each stage of the 100 decision trials. The dependent variable is the participant’s decision to invest or opt-out in any given trial.

##### 2.1.1.2. Procedure

The study was conducted as a laboratory experiment with each participant working alone and a maximum of 8 participants in any session. In each session, the participants first played the card game, and then demographic variables were assessed. Finally, the participants were debriefed, thanked, and paid 4 Euros and the performance-dependent bonus (potential range 0 to 7.80 Euros).

###### 2.1.1.2.1. VIP-task

**Figure 1:**
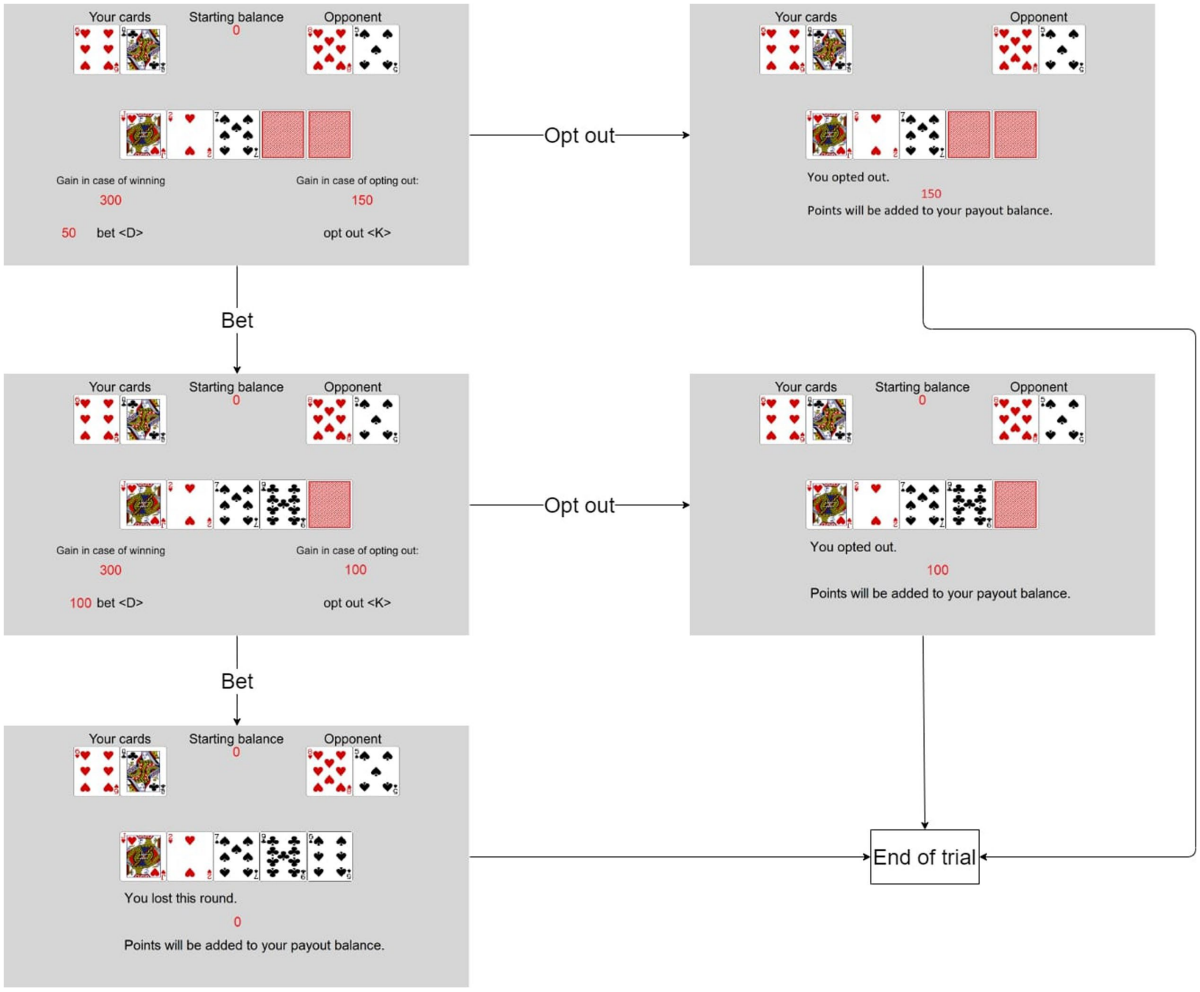
Example of a trial in the VIP-Task. This example starts with three shared cards revealed. A trial can have up to five stages if none of the shared cards are revealed at the start of the trial.

The VIP-Task was designed as a measure of incentivized sequential decisions in an uncertain and risky environment. It has previously been used to assess EoC (Doerflinger et al., 2017) and was implemented using PsychoPy (Peirce et al., 2019). The rules of the VIP-Task are based on the “Texas hold’em” variant of poker. In the VIP-Task, participants play against the computer (referred to as the opponent) and each trial contributes to the potential bonus. Trials are randomly generated for each participant. At the beginning of each trial, the participants had a fixed amount of points they could bet (i.e., invest) during the trial (see Figure 1 for an exemplary trial). Both the participant and the opponent have two individual cards in each trial (i.e., their hand). In addition, five shared cards can add to the value of both players’ hands. All cards are randomly drawn from a list of standard poker cards. Usually, all shared cards are hidden at the beginning of each trial. Participants decide whether to invest further or to opt out.

If they decided to invest, one of the hidden shared cards was revealed. The cost of investing in a trial increased with each revealed card. If they decided to opt-out, the current trial ended, and the remaining points were added to the participants’ payout. If participants invested until all shared cards are revealed, their cards were compared against their opponent’s by using standard poker rules. The value of the best five cards out of a player’s two individual cards and the five shared cards are compared to decide the winner. The five cards can be any combination of hand and shared cards. The points added to the payout depend on the outcome of this comparison: if the participant loses, no points are added; if the participant wins, twice the invested points are added; if the comparison ends in a tie, the invested points are added. After this comparison, the trial ends. At the end of each trial, a screen informs the participants of the results (i.e., win, lose, tie, opt out) and the points added to the payout.

Explicit probabilities were not shown to participants. We use the probability of losing (if the round is played until the end) as an independent variable ranging from 0 to 1. It is calculated based on the revealed cards. The probability of winning is complementary to it and using it produces the reversed pattern of results. Ties are rare and do not affect the overall results.

The participants were given a reference sheet explaining the standard poker rules. They could use the sheet throughout the experiment. After reading the rules of the game, the participants played three practice trials before the main task started. The task had 100 trials in total. Each trial had up to five stages in which the participants could invest up to 310 points, costing 10 points for the first investment, 20 points for the second, 40 points for the third, 80 points for the fourth, and 160 points for the fifth.

###### 2.1.1.2.2. Personal responsibility manipulation

In one half of the trials, the participants were personally responsible for each decision. In the other half, the trials started with some shared cards already revealed (between 1 and 4) and points invested accordingly. All trials were presented in random order. The trial stages after the participants had already made an investment decision themselves were coded as personally responsible. The trial stages directly following a computer-made investment decision were coded as assigned decisions, as the participants had no control over the invested points before their decisions. The first stage of a trial without revealed shared cards is not included because, at this stage, no prior investment had been made. Based on the assumption that when participants had made multiple prior investments, they shared more personal responsibility for any given situation, the stage of the trial corresponding to the number of prior investments (between 1 and 4) was included as a further indicator of the degree of responsibility.

#### 2.1.2. Results

A mixed effects logistic regression was used to predict the probability of investments. Independent variables were the probability of losing, personal responsibility, and the number of prior investments, as well as all their interaction terms. Random effects for participants and trial numbers were included. The model is summarized in Table 1. The main effect of the probability of losing, *z* = −8.62, *p* < 0.001, was significant, indicating a higher likelihood of reinvestments if the probability of losing was low. Personal responsibility, *z* = 3.32, *p* < 0.001, also had a significant main effect – participants were more likely to reinvest after decisions they were personally responsible for than after assigned decisions. Moreover, the number of prior investments, *z* = −6.30, *p* < 0.001, was a significant negative predictor of reinvestment, indicating that opting out was more prevalent in later than earlier stages of each trial. All two-way interaction effects were significant: the probability of losing and personal responsibility, *z* = −2.01, *p* = 0.045, the probability of losing and the number of prior investments, *z* = 2.58, *p* = 0.010, and personal responsibility and the number of prior investments, z = −2.27, *p* = 0.023. These effects were qualified by a significant three-way interaction effect between the probability of losing, personal responsibility, and the number of prior investments, *z* = 2.07, *p* = 0.038. For simple slope analyzes and Johnson Newman intervals see the supplemental materials.

**Table 1:** Mixed effects logistic regression estimating the decision to bet in Experiment 1.

Variable	OR	B	SE B	*z*	*p*
Intercept	492.75	6.20	0.46	13.37	<0.001
Probability of losing	0.003	−5.90	0.68	−8.62	<0.001
Personal responsibility^a^	10.18	2.32	0.70	3.32	<0.001
Prior investments	0.50	−0.69	0.11	−6.30	<0.001
Probability of losing × Personal responsibility^a^	0.13	−2.07	1.03	−2.01	0.045
Probability of losing × Prior investments	1.52	0.42	0.16	2.58	0.010
Personal responsibility^a^ × Prior investments	0.70	−0.36	0.15	−2.27	0.023
Probability of losing × Personal responsibility^a^ × Prior investments	1.62	0.48	0.23	2.07	0.038
Random effects (*s*^2^)	Participant: 0.35	Trial: <0.01			

^a^Assigned = 0, responsible = 1.

**Figure 2:**
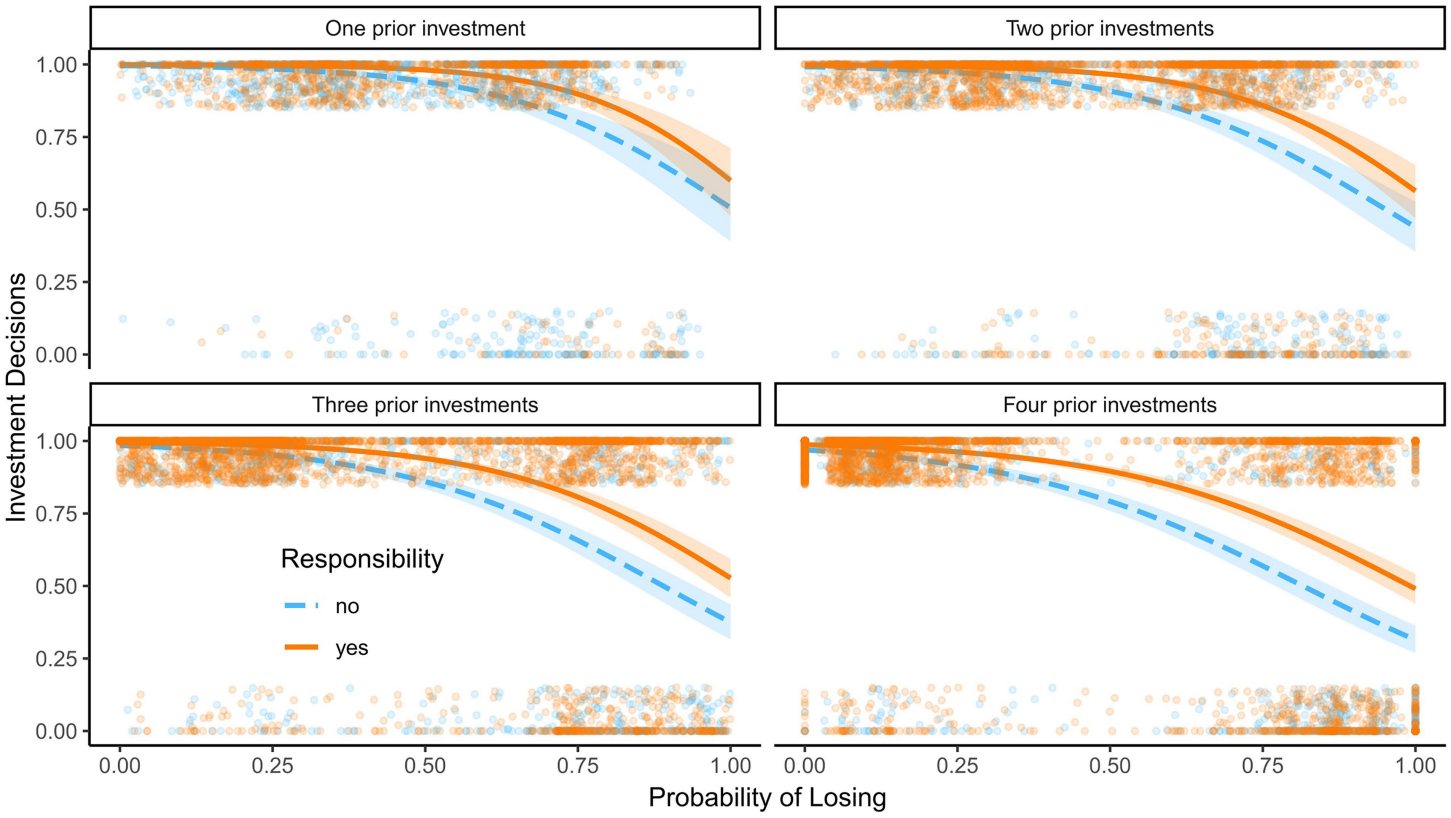
Reinvestments in Experiment 1. Predicted probability to reinvest as a function of the probability of losing, personal responsibility, and the number of prior investments in Experiment 1; 95% confidence intervals are displayed. Dots show raw data; the dots at the top are decisions to invest and the dots at the bottom are decisions to opt out.

The predicted probabilities are visualized in Figure 2. Participants were more likely to bet on a given hand at all levels of probability of losing when they were personally responsible for prior decisions. The difference between decisions for which the participants were personally responsible and assigned decisions was larger at higher probabilities of losing. Furthermore, the responsibility effect was more pronounced with multiple prior investments.

#### 2.1.3. Discussion

Participants made decisions in line with the rules of the task to increase their payout; they were more likely to reinvest if the probability of losing was low. Concerning personal responsibility, we found that participants were indeed more likely to reinvest in a hand if they were personally responsible for prior decisions, especially when the probability of losing was high. Note that we compare self-chosen and (random) computer chosen precursor decisions on the same level of success/failure probability; despite expecting self-chosen participant decisions to be more aligned to probabilities than the random computer decisions in general, comparing responses at the same probability levels removes this difference in the quality of decisions.

The smaller effects at low losing-probability levels are most likely the result of a ceiling effect, as both decisions for which the participants were personally responsible and assigned decisions are close to 100% continue/invest decisions. Our results are in line with previous evidence (Sleesman et al., 2012) that personal responsibility increases EoC. We observed larger personal responsibility effects at later stages of a trial where more prior investments had been made, indicating that the degree of personal responsibility is positively related to reinvestments.

There is a limitation, however: defining the degree of personal responsibility in terms of the number of prior investments is confounded with outcome uncertainty. Negrini et al. (2020) observed no responsibility effects on reinvestments in an incentivized experimental study. However, in Negrini et al.’s study both the cost of the initial investment and the likelihood that investments would ultimately lead to success were unknown to the participants when the initial investment was made. This very high degree of uncertainty might have undermined potential responsibility effects, because the decision makers could not estimate the quality of the first investment.

In a series of multiple reinvestments, each investment can also be understood as costly information search to reduce uncertainty and the sampled information can inform search decisions (Cohen and Erev, 2021). The more prior investments have been made, the more shared cards are revealed in the VIP-Task. While the risk of losing can increase or decrease with each investment, the new shared cards reveal previously unavailable information, thus reducing uncertainty. Some decision-makers may seek to reduce uncertainty or avoid investments in more uncertain situations (Nau, 2006).

The design of Experiment 1 does not allow for a fair evaluation based on the expected value of the participants’ decisions, because the expected value of decisions early in the sequence depends on the participants’ future decisions. Thus, additional evidence is needed to evaluate, when exactly personal responsibility is beneficial (to avoid prematurely opting out) or detrimental (EoC) to decision quality. To exclude uncertainty avoidance as an alternative explanation, and to allow an evaluation of the expected value, in Experiments 2 and 3, we only focused on the last decision in the sequence and modified the Poker task accordingly. That is, for all critical decisions, the same number of cards was revealed. The level of uncertainty was thus held constant.

Finally, the assigned decision trials were randomly generated decision situations with prior investments made by the computer. These situations are equivalent to decision situations following a sequence of arbitrary prior decisions. The participants were fully informed about the task procedure and the randomness of the assigned decision trials. Participants may have heuristically responded negatively to the random computer decisions. Thus, the personal responsibility effect could partially result from the negative connotation conveyed by knowing that the previous decision was made randomly. To account for the possibility of such a heuristic, we explicitly manipulated the quality of the assigned decisions in Experiment 2.

### 2.2. Experiment 2: Personal responsibility, preferences, and decision quality

To further test the personal responsibility hypothesis, we changed three aspects of the procedure: (1) We measured preferences to continue investing/opting out in the initial investment situation for each trial before the decision task. If we apply the arguments raised by Schulz-Hardt et al. (2009) to the results of Experiment 1, it seems possible that the responsibility effects we observed are due to the participants’ preferences for the initial card combinations. To account for this alternative explanation and also to test whether responsibility effects do occur in parallel to preference effects, we measured the participants’ preferences for each card combination that would be played (both for trials with and without personal responsibility). (2) In addition to assigned decision trials in which the initial decision was made randomly (equivalent to Experiment 1), we added assigned decision trials in which the initial decision was made optimally by the computer (based on the probability of losing). (3) The modified task also consisted of only one reinvestment decision per trial (at the last stage) after the initial investment decision. We thus avoided confounding uncertainty and responsibility. The expected value of each reinvestment decision made by the participants is now independent of future decisions, which allows us to analyze it as a dependent variable without taking into account the participants’ potential future decisions. We used the expected value of reinvestments as a plausibility check. The assigned optimal decisions should lead to a higher expected value than the assigned random decisions and participants should be more likely to indicate a preference for initial investments when the probability of losing is low. With only one critical reinvestment decision in the sequence, the probability of losing at which reinvesting is prudent can also be easily determined based on the expected value as a benchmark for EoC vs. prematurely opting out. Because the second investment costs twice as much as the first and the payout in case of winning is double the investment, the expected value for reinvesting is higher than for opting out, if the probability of losing is less than 2/3.

#### 2.2.1. Method

##### 2.2.1.1. Participants and design

Forty-nine participants (27 female) with a mean age of 23.7 (range 19 to 43, SD = 4.2) were recruited at a German university. Personal responsibility was manipulated within-participants as one of three trial types (personal responsibility: personally responsible vs. random assignment vs. optimal assignment). Preferences were measured as a dichotomous variable (preference vs. no-preference) for each trial before the card game started. Losing probability was calculated as a quasi-experimental predictor for each decision trial. The dependent variable is the participants’ decision to invest or opt out in any given trial. The study was conducted in the laboratory with up to 8 participants per session. The participants first indicated their preferences for each trial, then they played the card game, and thereafter provided demographic information. Finally, they were debriefed, thanked, and paid 5 Euros and a bonus dependent on the card game (potential range: 0–8.2 Euros).

##### 2.2.1.2. VIP-task

Standard poker rules were explained to the participants, and they were given a reference sheet that could be used during the experiment. In contrast to Experiment 1, all trials of the VIP-Task started with three of the five shared cards revealed. Therefore, in a standard trial, participants had to decide twice whether to invest or not – the first decision pertained to the initial investment and the second decision to the reinvestment. In each trial, 150 points could be invested. The first investment cost 50 points; the second investment cost 100 points. Decisions for the second investment are the dependent variable. Before the participants played the card game, we measured their initial preferences for each trial. The participants played 150 trials of the game, 50 trials for each within-participant condition.

###### 2.2.1.2.1. Preference measure

All trials were generated at the beginning of the experiment and randomly mixed. Before the card game was played, the participants were confronted with the initial investment situation (with three revealed shared cards) and asked whether they would hypothetically invest or not – this was done for each of the 150 trials presented in random order. This procedure allows for assessing initial preferences (as a dichotomous measure: preference vs. no preference) for all trials, including non-responsible trials.

###### 2.2.1.2.2. Personal responsibility manipulation

At the beginning of each trial, the participants saw their hand cards, the opponent’s hand cards, and three revealed shared cards. This was the same configuration that they had rated before on the preference task. Personally responsible trials played out the same way as in the standard VIP-Task: the participants decided whether to invest in the shown cards or not. If they invested, the fourth shared card was revealed, and they had to decide whether to reinvest or opt out. Each trial started with 150 points available to the participants to invest. If they opted out the first time around, the trial ended, and 150 points were added to their payout. If they opted out after having invested once, 100 points were added to their payout. If participants decided both times to invest in their cards, the fifth shared card was revealed, and the participants’ cards were compared to the opponent’s cards according to standard poker rules. Winning this comparison resulted in 300 points added to the participants’ balance; losing the comparison resulted in 0 points added, and if the participant and the opponent tied, 150 points were added.

**Figure 3:**
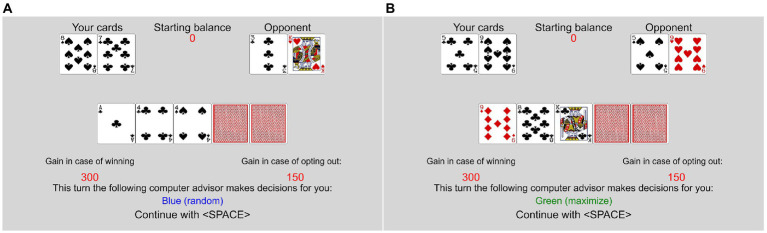
Examples of non-responsible trials in Experiment 2. **(A)** Random assignment, **(B)** optimal assignment.

Personal responsibility and the quality of prior decisions were varied by introducing two computer “advisors” to the participant. In some rounds, the participants played from the beginning, in others, one of the computer advisors made the initial investment decision. The advisors either invested points to reveal a hidden shared card or opted out. The participants always had to make the reinvestment decisions. Rounds in which the advisors opted out did not count toward the payout and were not counted as trials played. The rules to determine the winner for the trials were the same, whether the participants made the first decision or one of the advisors. In the random assignment trials, the computer would invest with a probability of 50% and opt out with a probability of 50%, independent of the card values. In the optimal assignment trials, the computer would invest when the participants’ chances of winning or a tie were better than the chances of losing. If the chances of losing were higher, the computer would opt out. If the advisors invested in the cards, a new shared card was revealed. This changed the chances of winning. See Figure 3 for examples of the assigned decision trials. The 5th and last card decision was then always decided by the participant.

The procedure was thoroughly described to the participants on the screen before the card game task started. The random assignment advisor was named “Random,” and the optimal assignment advisor was named “Maximize.” The advisors’ names were colored green versus blue (counterbalanced) to make it more intuitive to identify them. In the first stage of each random and optimal assignment trial, a message was displayed, indicating which advisor would make the first decision: “This time, the following computer advisor makes the first decision for you: Random/Maximize.”

###### 2.2.1.2.3. Expected value

Because the participants made only one reinvestment decision in each trial, the expected value of this decision could be calculated without further assumptions about future decisions. If participants opted out in the second decision, the expected value of that decision was 100 points. If they decided to invest, the expected value was calculated as 300 * (1-p_losing_).

#### 2.2.2. Results

##### 2.2.2.1. Expected value

A mixed linear model (full summary in supplemental materials) was calculated to predict the expected point value of the participants’ decision in each trial based on personal responsibility, preferences, and the interaction terms with random effects for participants. The main effect of preferences was significant, *t*(5066.7) = 18.92, *p* < 0.001. Compared to randomly assigned trials, the expected value was significantly higher in personally responsible trials, *t*(5065.2) = 8.29, *p* < 0.001, and in optimal assignment trials, *t*(5066.7) = 17.58, *p* < 0.001. These main effects were qualified by significant interaction terms of preference and personally responsible trials, *t*(5064.4) = −4.07, *p* < 0.001, and preference and optimal assignment trials, *t*(5070.4) = −7.97, *p* < 0.001. The difference between personally responsible, randomly assigned and optimally assigned trials was smaller in trials for which the participants had indicated a preference to invest.

##### 2.2.2.2. Reinvestment decision

We calculated a mixed effects logistic regression (summarized in Table 2) to predict investments based on the probability of losing, personal responsibility (dummy coded with random assignment trials as the baseline), and initial preferences. Interaction terms of the independent variables and random effects for participants were included. The main effect of the probability of losing, *z* = −14.06, *p* < 0.001, was significant, indicating that participants were more likely to invest when the probability of losing was low. The dummy coded personal responsibility variables also showed significant main effects, indicating that participants were more likely to invest in the optimal assignment trials, *z* = 2.40, *p* = 0.017, and the personally responsible trials, *z* = 7.73, *p* < 0.001, than in the random assignment trials. None of the interaction terms were significant, |*z*s| < 1.30, *p*s > 0.196.

**Table 2:** Mixed effects logistic regression estimating the decision to bet in Experiment 2.

Variable	OR	B	SE B	*z*	*p*
Intercept	4.02	1.39	0.17	8.15	<0.001
Probability of losing	0.04	−3.18	0.23	−14.06	<0.001
Trial type optimal^a^	1.67	0.51	0.21	2.40	0.017
Trial type responsible^a^	5.87	1.77	0.37	4.73	<0.001
Preference^b^	3.46	1.24	0.21	5.87	<0.001
Probability of losing × Trial type optimal^a^	1.27	0.24	0.45	0.54	0.591
Probability of losing × Trial type responsible^a^	0.75	−0.29	0.51	−0.57	0.568
Probability of losing × Preference^b^	0.66	−0.42	0.33	−1.29	0.196
Trial type optimal^a^ × Preference^b^	0.83	−0.19	0.29	−0.67	0.502
Trial type responsible^a^ × Preference^b^	0.73	−0.32	0.48	−0.65	0.515
Probability of losing × Trial type optimal^a^ × Preference^b^	0.68	−0.38	0.58	−0.65	0.514
Probability of losing × Trial type responsible^a^ × Preference^b^	0.64	−0.44	0.69	−0.64	0.523
Random effects (*s*^2^)	Participant: 0.25				

^a^Variables are dummy coded, random assignment trials are coded 0 on both variables; ^b^no-preference = 0, preference = 1.

A follow-up contrast test using a mixed effects logistic regression to compare only personally responsible trials versus optimal assignment trials with preferences as a control variable, and including random effects for participants, revealed in addition to the main effects of probability of losing, *z* = −30.44, *p* < 0.001, and preferences, *z* = 10.78, *p* < 0.001, a main effect of personal responsibility, indicating that participants were significantly more likely to reinvest in personally responsible trials than optimal assignment trials, *z* = 4.96, *p* < 0.001.

**Figure 4:**
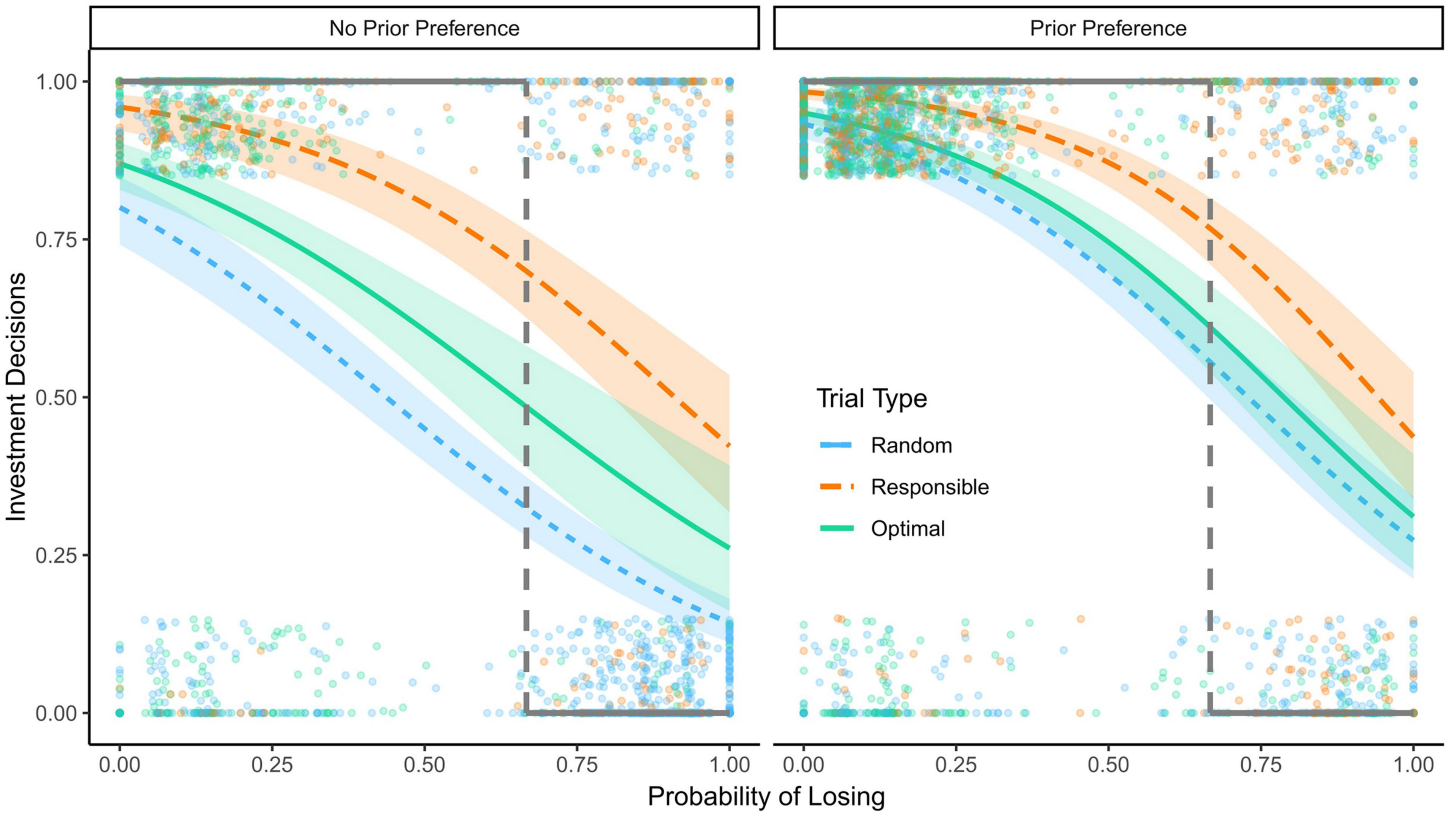
Reinvestments in Experiment 2. Predicted probability to reinvest as a function of the probability of losing, responsibility, and preferences in Experiment 2, 95% confidence intervals are displayed. Dots show raw data; the dots at the top are decisions to invest and the dots at the bottom are decisions to opt out. The dashed lines indicate the probability of losing at which reinvesting and opting out have the same expected value. The bold black lines show the hypothetical pattern of decisions that would yield the highest expected value.

The predicted probabilities are visualized in Figure 4. Participants were least likely to invest in their cards in trials in which the random advisor had made the first decision. Investments were more likely in trials in which the optimal advisor had made the first decision. The highest rate of reinvestments was in trials in which the participants had made the first decision themselves (i.e., personally responsible trials). These differences were unaffected by the probability of losing and participants’ initial preferences.

#### 2.2.3. Discussion

The expected value of the participants’ decisions was lowest in the random advisor trials. This is not surprising, as the initial decision quality of the random advisor is lower than that of both the participants and the optimal advisor. The expected value of the decisions in personally responsible trials was higher than in random assignment trials but still lower than in optimal assignment trials. Thus, the deviation induced by participants’ personal responsibility did lower their expected outcome compared to the optimal advisor.

The observed main effect of the probability of losing on reinvestments demonstrates that the participants understood the task they had to perform. They were more likely to keep investing in good cards than in bad cards. Preferences had an incremental effect on investments. Irrespective of the actual probability of losing, the participants were more likely to invest if they had indicated a preference for the initial decision, validating the preference measure and replicating prior evidence (Schulz-Hardt et al., 2009) that preferences are a factor in EoC. The effect of a preference for initial investments was present across all levels of the probability of losing – when the situation was unfavorable, an initial preference decreased decision quality (i.e., making EoC more likely), but when the situation was favorable, initial preferences increased decision quality (i.e., making prematurely opting out less likely).

We observed personal responsibility effects beyond measured preferences; responsibility remained a significant predictor even when controlling for preferences. Thus, we have some indication that preferences need to be considered but may not fully account for responsibility effects in reinvestment decisions. Personal responsibility made participants generally more likely to reinvest. At high probabilities of losing this effect resulted in decreased decision quality and at low probabilities of losing it increased decision quality. The dashed line in Figure 4 indicates the probability of losing at which reinvesting and opting out have the same expected value. To maximize the expected value, one should always invest in situations to the left of the line and always opt out in situations to the right (illustrated by the bold line in the figure). The confidence intervals for the participants’ investment decisions do not include the optimal strategy at any level of probability. The participants abandoned reinvestments too early, because the likelihood of reinvesting is below the optimal pattern for situations where reinvestment increases the expected value, even at a very low probability of losing. They also demonstrated EoC, because the likelihood of reinvesting was higher than the optimal pattern for situations where reinvestment decreased the expected value, even at a very high probability of losing.

Participants were also more likely to invest when the optimal advisor had made the initial decision rather than the random advisor. This pattern may be a reasonable heuristic based on the negative connotation of “random choices” in this context. However, the participants were even more likely to invest if they were personally responsible for the initial decision themselves compared to the optimal advisor. We found these effects controlling for preferences. As the participants knew that the optimal advisor maximized the expected value, more reinvestments after their own initial decisions over the optimal advisor’s is a deviation from a normative expected utility perspective.

### 2.3. Experiment 3: Personal responsibility and framing effects

According to prospect theory accounts of EoC (Thaler, 1980; Whyte, 1986; Soman, 2004), loss framing should increase escalation behavior. Decision makers might construe invested resources as potential losses and seek to minimize them by making further risky investments. This however constitutes a risky option: failure means that the second investment will also be lost, whereas success could mitigate the loss of the investments made. Accordingly, we hypothesized that participants would be more likely to invest if the task is presented in a loss frame than a gain frame. Using incentivized decisions with real feedback goes beyond the past research on framing effects on EoC, which relied on hypothetical investment scenarios. We found such framing effects in a pilot study that followed a procedure similar to Experiment 1; we manipulated whether the outcomes were presented as gains or losses (see supplemental material). Based on this pilot study, in Experiment 3, we combined the three determinants of EoC: personal responsibility, preferences, and gain vs. loss framing. We also included the numeracy scale (Lipkus et al., 2001) and the gambling and investing risk-taking propensity subscales of the DOSPERT scale (Weber et al., 2002) as individual difference measures. Numeracy might be beneficial to participants when judging the probability of losing, while a general disposition for risk-taking might make reinvestments more likely in the present task. The experiment was preregistered on osf.org: https://osf.io/zg7xm/?view_only=b913923de9ce4dccbddd6929678a398c.

#### 2.3.1. Method

##### 2.3.1.1. Participants and design

Eighty participants (23 female) with a mean age of 23.3 (range 18–46, SD = 5.0) were recruited online *via* prolific.org (Palan and Schitter, 2018). The sample included participants from Europe, Asia, Northern America, and Middle America. The experiment had a 3-within (personal responsibility: personally responsible vs. random assignment vs. optimal assignment) by 2-between (framing: gain vs. loss) design, with preferences as an additional independent variable measured for each trial. Probability of losing was calculated for each trial as a quasi-experimental factor. The dependent variable is the participants’ decision to invest or opt out in any given trial.

##### 2.3.1.2. Procedure

The experiment was conducted as an online experiment using the JavaScript functions of PsychoPy (Peirce et al., 2019) on the pavlovia.org platform. We asked participants first to fill out the numeracy scale (Lipkus et al., 2001) and the gambling and investing risk-taking propensity subscales of the DOSPERT scale (Weber et al., 2002). Then we measured preferences in the same way as in Experiment 2. Finally, the participants played the VIP-Task. They were paid 5 GBP and a bonus dependent on the card game (potential range: 0–5.4 GBP). Demographic information was obtained from prolific.org.

###### 2.3.1.2.1. VIP-task

The participants played 90 trials of the VIP-Task, which was structured in line with Experiment 2. Personal responsibility was manipulated the same way: in one third of the trials a random advisor made the first investment, in another third an optimal advisor made the first investment, and in the final third the participants made the first investment decision. The VIP-Task was presented with outcomes framed either in terms of gains or losses. Participants could display the Poker rules on the screen by pressing a key at any time during the experiment.

###### 2.3.1.2.2. Framing

**Figure 5:**
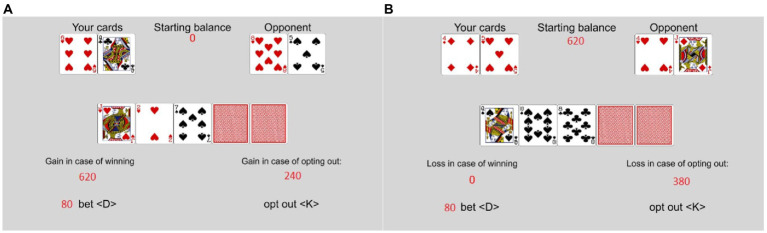
Example of responsible trials in Experiment 3. **(A)** Gain frame, **(B)** loss frame.

See Figure 5 for an example of trials in the gain and loss frame conditions. In the *gain frame* condition, participants were informed that they started the game with 0 points and could gain between 0 and 300 points each turn. The maximum gains were 27.000 points in total. In the *loss frame* condition, the participants were informed that they started the game with 27.000 points and could lose between 0 and 300 points each turn. The maximum losses were 27.000 points. The game played out the same way, and the bonus was calculated the same way in the two framing conditions. The only difference was in the presentation as gains versus losses. Five thousand points were equivalent to 1 GBP, to be paid rounded mathematically to one penny. In the gain frame condition, the optimal advisor was named “maximize,” in the loss frame condition it was named “minimize.” In each trial, the participants’ starting balance for the trial was displayed in the top center of the screen. This was 0 in the gain frame condition and 300 in the loss frame condition. Below the shared cards, the gains/losses, respectively, in case of winning the turn were shown and the gains/losses in case of opting out. In the gain frame condition, the results screen included “### Points are added to your payout balance,” and in the loss frame condition this text read “### Points are subtracted from your payout balance.”

#### 2.3.2. Results

##### 2.3.2.1. Expected value

We calculated a mixed linear model to predict the expected value of the participants’ decisions based on framing, preference, personal responsibility (dummy coded with random assignment trials as the baseline), and their interaction terms. Numeracy and risk taking were included as covariates, and random effects were used for participants (see supplemental materials for full summary). This analysis with the expected value as dependent variable was exploratory as it was not specified in the preregistered analysis plan.

Neither the main effect of framing nor any of the interaction terms including framing were significant, |ts| < 1.05, ps > 0.260. Preferences, *t*(6659.6) = 14.29, *p* < 0.001, optimal assignment trials, *t*(3433.2) = 12.68, *p* < 0.001, and personally responsible trials, *t*(2030.6) = 2.86, *p* = 0.004, showed significant main effects. The interaction term of optimal assignment trials and preferences was also significant, *t*(6650.1) = −5.05, *p* < 0.001. In line with Experiment 2, the expected value in optimal assignment trials was higher than in random assignment trials and personally responsible trials. This difference was smaller in trials for which the participants had indicated a preference. In addition, numeracy was a significant positive predictor of the expected value, *t*(73.3) = 2.28, *p* = 0.026, while the risk-taking score was not, *t*(82.57) = 0.13, *p* = 0.897.

##### 2.3.2.2. Reinvestment decision

A mixed model using the same predictors as in Experiment 2 – probability of losing, personal responsibility (dummy coded with random assignment trials as the baseline), initial preferences, and the interaction terms of these variables replicates the pattern of results found in Experiment 2. Because the addition of Experiment 3 is the framing manipulation, we focus here, as pre-registered, on analyzes including main effects and interaction terms of framing, treating preferences for prior investments as a covariate.

**Figure 6:**
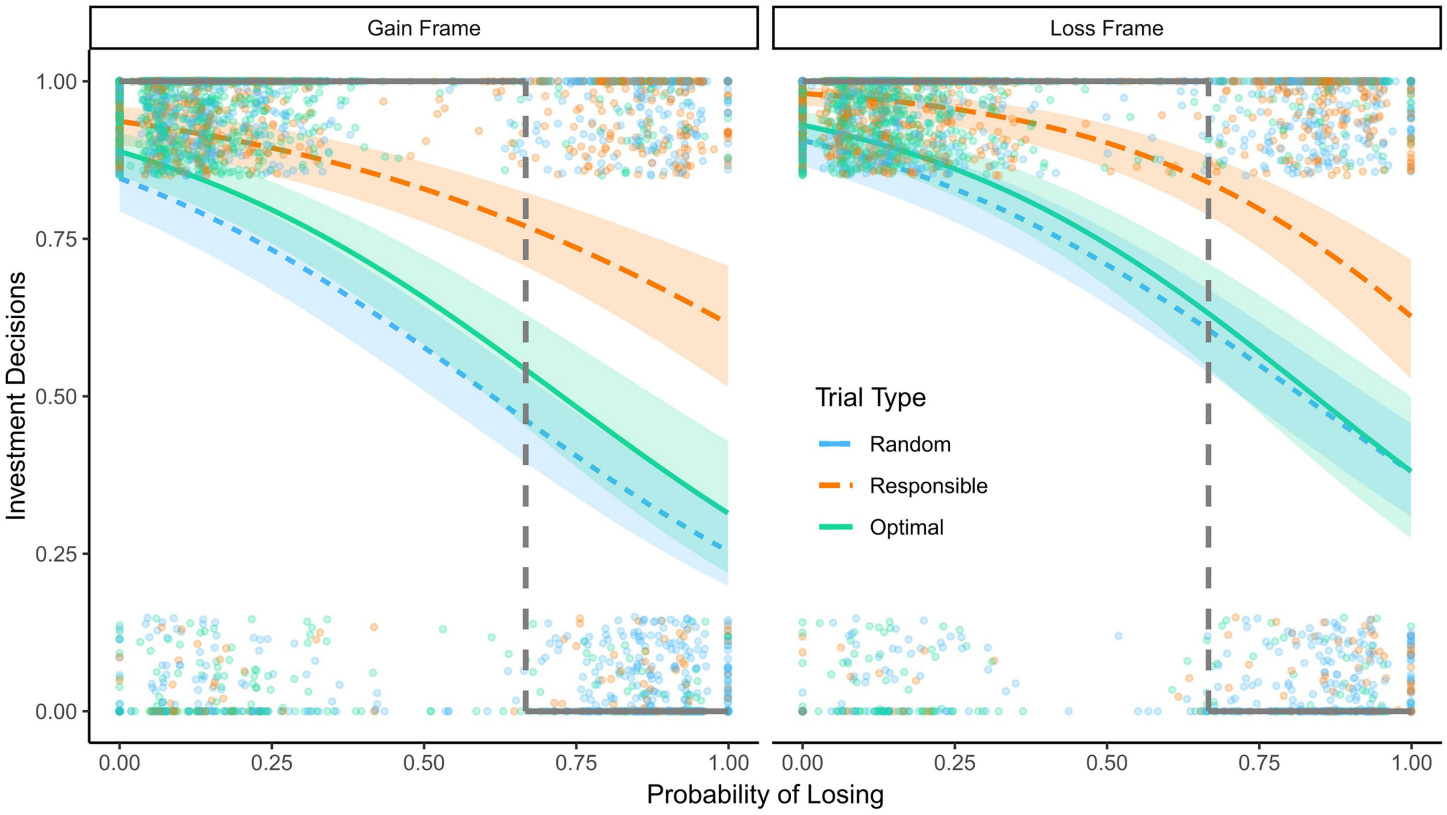
Reinvestments in Experiment 3. Predicted probability to reinvest as a function of the probability of losing, responsibility, and framing in Experiment 3, 95% confidence intervals are displayed. Dots show raw data; the dots at the top are decisions to invest and the dots at the bottom are decisions to opt out. The dashed lines indicate the probability of losing at which reinvesting and opting out have the same expected value. The bold black lines show the hypothetical pattern of decisions that would yield the highest expected value.

The probability of investments was predicted with a mixed effects logistic regression. As independent variables, we included the probability of losing, personal responsibility, initial preferences, framing, and the two-way and three-way interactions of the probability of losing, personal responsibility, and framing. Random effects are included for participants. The model is summarized in Table 3 and the predicted probabilities are illustrated in Figure 6. The main effect of the probability of losing was significant, *z* = −14.11, *p* < 0.001, indicating a higher probability of investments when the probability of losing was low. Compared to random assignment trials, the participants were significantly more likely to invest in optimal assignment trials, *z* = 2.07, *p* = 0.039, and even more likely in personally responsible trials, *z* = 3.82, *p* < 0.001. We found a framing effect, *z* = 2.06, *p* = 0.039, indicating that participants in the gain frame condition were less likely to invest than participants in the loss frame condition. The participants were also more likely to invest in trials for which they had indicated a preference before the task, *z* = 9.56, *p* < 0.001. None of the two-way interactions reached significance, |zs| < 1.54, ps > 0.124, but the three-way interaction of the probability of losing, personally responsible trials, and framing was significant, *z* = −1.99, *p* = 0.047. The Numeracy and the DOSPERT scale were no significant predictors, |zs| < 0.0.85, ps > 0.39. For simple slope analyzes and Johnson Newman intervals see the supplemental materials.

**Table 3:** Mixed effects logistic regression estimating the decision to bet in Experiment 3.

Variable	OR	B	SE B	*z*	*p*
Intercept	139.77	2.40	0.76	3.14	0.001
Probability of losing	0.06	−2.78	0.20	−14.11	<0.001
Trial type optimal^a^	1.45	0.37	0.18	2.07	0.039
Trial type responsible^a^	2.69	0.99	0.26	3.82	<0.001
Preference^b^	2.10	0.74	0.08	9.56	<0.001
Framing^c^	1.72	0.54	0.26	2.06	0.039
Numeracy	0.94	−0.06	0.07	−0.85	0.396
DOSPERT	0.94	−0.06	0.14	−0.42	0.672
Probability of losing × Trial type optimal^a^	0.93	−0.07	0.35	−0.22	0.828
Probability of losing × Trial type responsible^a^	1.77	0.57	0.37	1.54	0.125
Probability of losing × Framing^c^	0.99	−0.01	0.30	−0.04	0.967
Trial type optimal^a^ × Framing^c^	0.99	−0.01	0.28	−0.03	0.978
Trial type responsible^a^ × Framing^c^	1.95	0.67	0.44	1.52	0.130
Probability of losing × Trial type optimal^a^ × Framing^c^	0.77	−0.26	0.51	−0.53	0.598
Probability of losing × Trial type responsible^a^ × Framing^c^	0.31	−1.18	0.60	−1.99	0.047
Random effects (*s*^2^)	Participant: 0.25				

^a^The variables are dummy coded, random assignment trials are coded 0 on both variables; ^b^no-preference = 0, preference = 1; ^c^gain = 0, loss = 1.

The participants were more likely to invest in their cards in optimal assignment trials than in random assignment trials, but they were even more likely to invest in personally responsible trials. The figure also shows that the three-way interaction resulted in a smaller difference between assignment trial types (i.e., random assignment vs. optimal assignment advisor) and between the assignment trials and the personally responsible trials in the loss frame condition compared to the gain frame condition. This seems to be a consequence of the higher likelihood of participants in the loss frame condition to invest in the assignment trials, particularly when the probability of losing was high.

#### 2.3.3. Discussion

##### 2.3.3.1. Plausibility check

Framing had no significant effect on the expected value of the decisions. A likely reason for this null-effect is that participants were more likely to reinvest in the loss frame than the gain frame condition irrespective of the probability of losing. Across the probability range, the costs and benefits of the framing conditions canceled each other out. For the remaining effects the same pattern of results for preferences and personal responsibility as in Experiment 2 were observed. The expected value was highest in optimal assignment trials and lowest in random assignment trials, with personally responsible trials sitting in-between. Preferences were a positive predictor of expected value. The difference between trial types was smaller for trials that the participants preferred. This pattern shows that preferences were adequately measured and the trial types worked as intended.

Participants were more likely to invest in bad cards, when they were personally responsible for prior investments, than when they were not. They were also less likely to invest in good cards when dealing with assigned decisions (see Figure 6). Both of these trends observed on the level of decision-making lead to a suboptimal expected value of the reinvestment decisions, reflecting the two types of decision errors investigated in the present research.

Numeracy positively predicted the expected value, but risk taking did not. This is plausible because a risk main effect can yield better outcomes in some trials and worse ones in others. Being better able to accurately judge the probability of losing, which is related to numeracy, is likely beneficial to performing well on the task at hand – further indicating the task’s validity. In contrast, a general preference for risky or safe options is neither an advantage nor a disadvantage.

##### 2.3.3.2. Responsibility, decision quality, preferences, and framing

The present study replicated the central findings from Experiments 1 and 2. Thereby, we provide another replication of personal responsibility effects beyond preferences. Preference effects are observed parallel to framing effects. Framing effects were evident, as participants were more likely to bet in a loss than a gain frame. Besides these central findings, somewhat surprisingly, the personal responsibility-framing interaction is not in line with the findings reported by Rutledge (Rutledge, 1995). Personal responsibility effects were not enhanced in the loss-frame condition; actually; there was a smaller difference between the personal responsibility conditions in the loss framing condition. A possible explanation is that participants in the loss frame condition were more likely to bet in assignment trials than participants in the gain frame condition, especially when the probability of losing was high. In personally responsible trials, the participants were more likely to bet both in the gain as well as the loss frame condition compared to the assigned trials, but the probability to reinvest did not differ between framing conditions for the responsible trials. This led to a smaller difference between personally responsible and assignment trials in the loss condition than the gain condition, which resulted in the observed interaction effect.

The probability to reinvest for personally responsible trials was not diminished in the loss frame condition; instead, participants in the loss frame condition were more likely to reinvest in assigned trials than participants in the gain frame condition. This pattern may be driven by a ceiling effect for the personally responsible trials. Although the personally responsible decisions were not close to the actual ceiling of 100%, there is probably a limit to participants’ mindlessly continuing at very high probabilities of losing. With a lower starting point for the assigned trials, there was more room to be pushed toward risky investing caused by loss framing. Also, there may generally be a limit to the degree of escalation (i.e., reinvestment at high losing probabilities) that can be expected for any given decision problem, and personal responsibility effects may be sufficient to push decision makers to that limit. Both construing the situation as a choice between losses and being personally responsible for prior decisions increase the probability of investments, but this does not mean that decision makers will completely disregard available information about the likelihood of success or failure.

## 3. General discussion

Decisions to continue with a previously chosen course of action or to quit can be critical for individuals (financially, personally) and even societies (e.g., when to continue or withdraw regulations to contain an ebbing pandemic). As such decisions can be highly consequential, it is important to understand psychological factors that can influence them. Different determinants of reinvestment decisions have been proposed in the literature on escalation of commitment (EoC) and some have been questioned (e.g., Schulz-Hardt et al., 2009). We argued that the determinants were often tested in hypothetical scenarios and by using bogus feedback. This is a critical limitation as anticipated responses do not necessarily line up with responses made in the actual situation (Nordgren et al., 2009). Other researchers agree with this assessment (Roth et al., 2015; Negrini et al., 2020). Even more, a recent study (Negrini et al., 2020) showed that determinants of reinvestment (amount of prior investment) can differently affect hypothetical and financially consequential decisions. They observed that higher prior investments led to escalation of commitment in hypothetical scenarios, but a reverse effect in the incentivized task. The authors also varied responsibility for previous investments. They found no effect of responsibility on reinvestments in their incentivized task. These findings, which are seemingly inconsistent with the EoC literature, suggest that additional experiments using incentivized tasks are needed to probe whether responsibility effects occur when real money is on the line.

We use a behavioral decision task with real financial consequences (Doerflinger et al., 2017). We validated the previously proposed responsibility and framing factors, finding evidence for their effects even when controlling for alternative explanations (preferences). When comparing with objectively (i.e., probability based) optimal decisions, our studies indicate that the presence of these factors (responsibility or loss framing) are not only relevant for continuing to invest beyond what is optimal, but that their absence can also lead to dropping out earlier than what is optimal.

### 3.1. Personal responsibility and preferences for the initial investment

Schulz-Hardt et al. (2009) observed in two studies that personal responsibility effects on EoC disappeared after statistically controlling for initial preferences. In our studies, preferences increased the probability of continuing investing, irrespective of success versus failure. However, preferences did not impact the influence of personal responsibility on reinvesting in the present studies. Instead, personal responsibility and preferences had an additive effect on the likelihood of reinvesting both in unfavorable situations (i.e., escalation of commitment) and favorable situations (i.e., avoiding prematurely opting out).

### 3.2. Personal responsibility and framing

According to the prospect theory account of EoC, framing in terms of gains or losses should influence the degree of escalation of commitment (Soman, 2004). In Experiment 3, the likelihood of reinvestments was lower when outcomes were framed as gains compared to losses. In line with the hypothesis that loss framing is a relevant factor in driving reinvestments, gain framing decreased reinvestments. In Experiment 3, where framing and personal responsibility were varied, framing moderated the effect of personal responsibility. A loss frame increased the probability of reinvesting for trials without personal responsibility for the initial decision; the difference between personally responsible and assignment trials was smaller in the loss frame condition than in the gain frame condition.

Based on Rutledge (Rutledge, 1995), we predicted that loss framing should have magnified responsibility effects as decision makers should be even more hesitant to lose something based on their own prior decision. However, we did not observe such an effect. This might be due to a ceiling effect of the already high probability of continuing to invest when participants were personally responsible so that there was less room for loss framing to drive this further. Beyond this unexpected effect, however, we can conclude that framing has an effect on reinvestments overall.

### 3.3. Decision quality

Escalation of commitment is often referred to as a decision bias, implying that it is not rational (Brockner, 1992) and therefore a suboptimal strategy. While EoC is unprofitable from an economic perspective, one may argue from a preference perspective that sticking to a chosen course of action is “rational” because it matches one’s preferences (Schulz-Hardt et al., 2009). We found evidence that personal responsibility increased reinvestments beyond what would be expected solely based on the preference for the initial decision.

Furthermore, in Experiments 2 and 3, we found that participants were more likely to keep investing if they were personally responsible for prior decisions compared to both assigned decision mechanisms – assigned decisions made randomly and systematically (i.e., following the expected value principle). This effect was observed in both experiments for trials where participants had indicated a prior preference and for trials where they had not. The pattern of results can thus not solely be attributed to overestimating the quality of one’s initial decision because participants were more likely to reinvest when they were personally responsible, even compared to trials for which they knew that the computer had made an optimal initial decision. Responsibility effects also held while controlling for preferences regarding the initial decisions.

The expected value of the participants’ decisions was lower when the initial decision was made by the participants themselves rather than made optimally by the computer. As the probability of investing was consistently higher in the personally responsible condition than in the optimal assignment condition at all probability levels, this difference in expected value is driven by participants’ tendency to bet too much on bad hands for which they were personally responsible – which can be expected as a result of personal responsibility effects in EoC.

From an expected-value perspective, the participants reinvested too often in bad cards if they were personally responsible for the initial decision, and they reinvested not often enough in good cards if the initial decision was not their responsibility. Participants were more likely to reinvest at a high probability of losing in responsible trials compared to the two assignment conditions (see Figures 4, 6). They were also less likely to reinvest at a low probability of losing in the assignment trials than in responsible decision trials. Too many bad reinvestments lower the expected value in the personally responsible condition, and too few good reinvestments lower the expected value in the two assignment conditions, compared to a hypothetical decision strategy that would maximize the expected value (the bold lines in Figures 4, 6). In conclusion, the sum of our results indicates that being personally responsible interfered with optimal subsequent decisions (even beyond preferences) in those situations where the probability of success was low. When the probability of success was high however, personal responsibility was beneficial as it increased the likelihood of (good) reinvestments. This reasoning can also be applied to loss framing – being in a loss frame increases the probability to reinvest. In situations with a high probability of success this can be advantageous, but in situations with a high probability of failure it can be disadvantageous.

### 3.4. Variable investment poker task

The incentivized poker-based task used in the present experiments has several advantages over standard EoC paradigms such as hypothetical investment scenarios (e.g., Staw, 1976; Garland and Newport, 1991; Schulz-Hardt et al., 2009; Feldman and Wong, 2018; Benschop et al., 2020; Lee et al., 2020), or paradigms using deception (e.g., Strube and Lott, 1984; Schulz-Hardt et al., 2009). The rules of the task are fully and transparently described to the participants. Trials are generated randomly following these rules. This procedure created realistic decision problems, in some of which further investments yield a better outcome, while in others opting out results in higher payoffs. The participants’ decisions can thus be analyzed in relation to normative decision theories (e.g., expected utility theory; Von Neumann and Morgenstern, 1944). From a procedural validity perspective, the participants in the VIP task know that their decisions are consequential compared to hypothetical scenarios where they make decisions while knowing that they merely pretend to be the CEO of a million-dollar company. Besides the issues with anticipated versus real decisions, outcomes of hypothetical scenarios may also be biased by participants not anticipating their own decisions but by what they anticipate what a CEO would or should do. Such problems are avoided in the task paradigm used in our present research.

Anecdotal feedback by our participants suggests that the task is highly engaging and holds the participants’ interest and attention in the lab and online over even a large number of trials. The present task paradigm consists of multiple repeated trials, as opposed to one or two scenarios. This increases statistical power and allows within-participant manipulations of relevant factors. In the present line of studies, personal responsibility was manipulated within participants, while framing was manipulated between participants. But both variables could also be manipulated in a within-participants design using the VIP-Task. Materials to implement the task are available at https://osf.io/hdczr/?view_only=546aec80e6f7468685072d199a9d9821.

## 4. Conclusion and outlook

Being personally responsible for prior decisions and loss framing increased the likelihood that decision makers reinvested in an ongoing course of action. These effects were robust and occurred beyond preferences. In situations where the probability of failure was high, personal responsibility, preferences and loss framing decreased the expected value of the reinvestment decisions, but in situations where the probability of failure was low, these variables increased the expected value. A comparison to optimal assigned initial decisions indicates that decision quality suffered when decision-makers were personally responsible for prior investments; in particular, when they were personally responsible for prior decisions they were more likely to throw good money after bad.

The card-game task used in our experiments is a powerful tool for future research. For example, it could be adopted for investigating social decisions. In our studies, advisors were computer algorithms. This has the advantage that the decision rules for these advisors can be exactly determined. However, it will be interesting to investigate whether real human advice is treated differently. It would also be interesting to analyze cooperative or competitive decisions with a modified VIP-Task, in which participants’ decisions affect the payout of other players.

## Data availability statement

The datasets presented in this study can be found in online repositories. The names of the repository/repositories and accession number(s) can be found in the article/Supplementary material.

## Ethics statement

The studies involving human participants were reviewed and approved by Ethik-Kommision, Universtiy of Konstanz. The patients/participants provided their written informed consent to participate in this study.

## Author contributions

JD created the experimental task, analyzed the data, and wrote the initial draft of the manuscript with input from TM-H and PG. TM-H and PG contributed additional written sections to the manuscript and provided guidance and editorial feedback. JD, TM-H, and PG involved in the conceptual development of the studies and the final preparation of the manuscript. All authors contributed to the article and approved the submitted version.

## Funding

The authors gratefully acknowledge financial support from the Ausschuss für Forschungfragen, University of Konstanz.

## Conflict of interest

The authors declare that the research was conducted in the absence of any commercial or financial relationships that could be construed as a potential conflict of interest.

## Publisher’s note

All claims expressed in this article are solely those of the authors and do not necessarily represent those of their affiliated organizations, or those of the publisher, the editors and the reviewers. Any product that may be evaluated in this article, or claim that may be made by its manufacturer, is not guaranteed or endorsed by the publisher.
