# Supplementary material for: Exploring the determinants of reinvestment decisions: Sense of personal responsibility, preferences, and loss framing
Source: Front Psychol. 2023 Jan 12;13:1025181. doi: 10.3389/fpsyg.2022.1025181 (PMC9878561; doi:10.3389/fpsyg.2022.1025181)
Supplement: Supplementary file 1 [file Table_1.DOCX]

Supplementary Material

# Experiment 1

## Conditional Effect of Responsibility

**Supplementary Table 1**

*Simple slope analyses in Experiment 1, conditional slopes and intercepts of Responsibility.*

|  |  |  | *B* | *SE B* | *z* | *p* |
| --- | --- | --- | --- | --- | --- | --- |
| PI ^a^ = 1 | POL^b^ = .138 (- 1*SD*) | Conditional intercept | 5.11 | 0.20 | 25.64 | <.001 |
|  |  | Responsibility | 1.45 | 0.32 | 4.54 | <.001 |
|  | POL^b^ = .447 (*M*) | Conditional intercept | 3.31 | 0.13 | 26.22 | <.001 |
|  |  | Responsibility | 1.11 | 0.17 | 6.69 | <.001 |
|  | POL^b^ = .755 (*+ 1SD*) | Conditional intercept | 1.52 | 0.11 | 13.79 | <.001 |
|  |  | Responsibility | 0.77 | 0.13 | 5.86 | <.001 |
| PI ^a^ = 2 | POL^b^ = .138 (- 1*SD*) | Conditional intercept | 4.28 | 0.14 | 29.79 | <.001 |
|  |  | Responsibility | 1.15 | 0.21 | 5.61 | <.001 |
|  | POL^b^ = .447 (*M*) | Conditional intercept | 2.71 | 0.10 | 29.79 | <.001 |
|  |  | Responsibility | 0.96 | 0.11 | 9.04 | <.001 |
|  | POL^b^ = .755 (*+ 1SD*) | Conditional intercept | 0.77 | 0.08 | 12.11 | <.001 |
|  |  | Responsibility | 1.15 | 0.10 | 9.26 | <.001 |

*Note.* ^a^ prior investments, ^b^ probability of losing.

**Supplementary Table 1**

*Simple slope analyses in Experiment 1, conditional slopes and intercepts of Responsibility.*

|  |  |  | *B* | *SE B* | *z* | *p* |
| --- | --- | --- | --- | --- | --- | --- |
| PI ^a^ = 3 | POL^b^ = .138 (- 1*SD*) | Conditional intercept | 3.45 | 0.11 | 32.11 | <.001 |
|  |  | Responsibility | 0.86 | 0.13 | 6.72 | <.001 |
|  | POL^b^ = .447 (*M*) | Conditional intercept | 2.12 | 0.09 | 23.10 | <.001 |
|  |  | Responsibility | 0.82 | 0.07 | 11.14 | <.001 |
|  | POL^b^ = .755 (*+ 1SD*) | Conditional intercept | 0.78 | 0.09 | 12.48 | <.001 |
|  |  | Responsibility | 0.78 | 0.06 | 8.80 | <.001 |
| PI ^a^ = 4 | POL^b^ = .138 (- 1*SD*) | Conditional intercept | 2.63 | 0.11 | 23.50 | <.001 |
|  |  | Responsibility | 0.56 | 0.16 | 3.62 | <.001 |
|  | POL^b^ = .447 (*M*) | Conditional intercept | 1.52 | 0.10 | 15.92 | <.001 |
|  | x | Responsibility | 0.67 | 0.10 | 6.86 | <.001 |
|  | POL^b^ = .755 (*+ 1SD*) | Conditional intercept | 0.41 | 0.09 | 4.40 | <.001 |
|  |  | Responsibility | 0.78 | 0.09 | 8.71 | <.001 |

*Note.* ^a^ prior investments, ^b^ probability of losing.


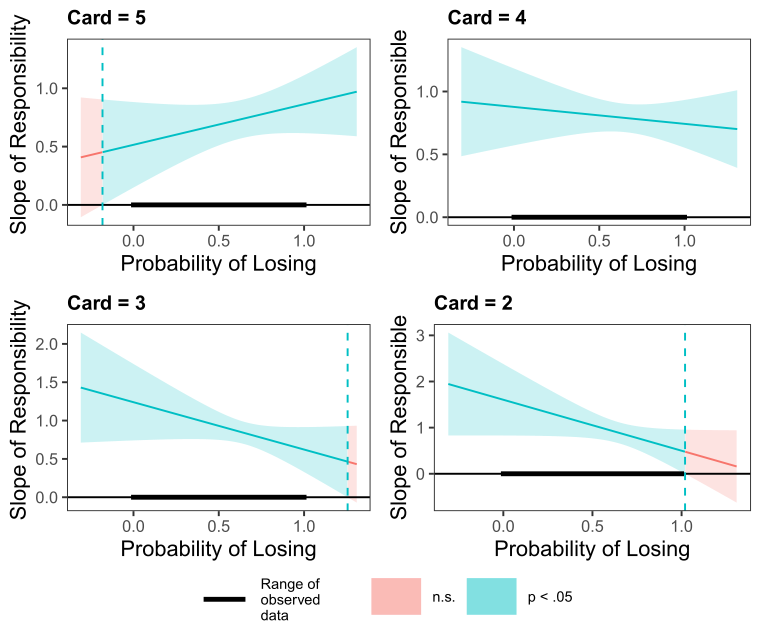


**Supplementary Figure 1**

*Johnson-Newman intervals for the conditional effect of responsibility on reinvestments in Experiment 1 with the number of prior investments and the probability of losing as the moderators.*

# Experiment 2

## Predicting Expected Value

**Supplementary Table 2**

*Mixed linear model predicting the expected point value based on trial type and preferences in Experiment 2.*

| Predictors | *B* | *CI* | *p* |
| --- | --- | --- | --- |
| (Intercept) | 130.76 | 124.25 – 137.27 | <.001 |
| Trial type responsible a | 43.93 | 33.54 – 54.32 | <.001 |
| Trial type optimal a | 77.75 | 69.08 – 86.42 | <.001 |
| Preference b | 71.11 | 63.74 – 78.48 | <.001 |
| Trial type responsible a × Preference b | -26.60 | -39.42 – -13.78 | <.001 |
| Trial type optimal a × Preference b | -44.48 | -55.42 – -33.54 | <.001 |
| Random Effects (*s^2^*) | Participant: 150.1 | | |

*Note.* ^a^ The variables are dummy coded, random assignment trials are coded 0 on both variables; ^b^ no-preference = 0, preference =1.

# Pilot Study: Framing Pilot

## Method

### Participants and Design

Seventy-one individuals (55 female) with a mean age of 23.2 (range 18 – 43, *SD* = 3.9) recruited at a German University took part in the study. According to the power analysis, the statistical power to detect small effects (*OR* = 1.4) was *β*‑1 > .95 at the α = .05 significance level in a mixed linear logit model. The power estimation was done the same way as in Experiments 1 and 2. Framing (gain vs. loss) was manipulated between participants with two versions of the task that were identical in content but different in regards to whether the points in each round of the task were presented as a potential gain (participants started with nothing and received points in each round) or a potential loss (participants started with the full amount of points on their account and lost points in each round). The probability of losing in any given trial was a quasi-experimental predictor variable. The dependent variable is the participants’ decisions to invest additional money into a round or opt-out. The study was conducted at the laboratory with up to 8 participants per session. The participants were paid 4 Euros and an additional payment dependent on the points earned in the VIP-Task (potential range: 0 to 8 Euros).

At the beginning of each session, the participants’ knowledge of poker was tested. Then they were randomly assigned to the gain or loss condition and played the respective version of the VIP-Task. After completing the card game task, the participants provided demographic information and indicated how many times they had played poker in the last month. Finally, the participants were thoroughly debriefed, thanked, and paid.

### Procedure

The main task had 80 trials. Gain and loss versions of the VIP-Task worked the same way, but the information was presented differently according to the framing condition. As in Experiment 1, each trial had up to five stages. Participants had to decide whether to invest points to reveal one of the five shared cards or opt-out at each stage. The participant could invest up to 310 points in each trial. The first investment cost 10 points, the second 20 points, the third 40 points, the fourth 80 points, and the fifth 160 points.

#### Framing manipulatio

Three texts were used on the screen to vary the framing of the task. First, in the upper center of the screen, a reminder was displayed. In the gain frame condition, it read: “You start this round with 0 points.” In the loss frame condition, it read, “This round is about 620 points.” Second, gains/losses in case of winning were shown below the shared cards on the left side of the screen. In the gain frame condition, the text read: “Gain in case of winning: 620.” In the loss frame condition, the text read: “Loss in case of winning: 0.” Gains/losses in case of opting out were shown below the shared cards on the right side of the screen. The text in the gain frame condition was “Gain in case of opting out: ##,” and in the loss frame condition “Loss in case of opting out: ##,” with the respective gains/losses depending on the stage of the trial. *Third*, the last screen of each trial showed in addition to information about the choices and the outcome (i.e., win, lose, draw, opt-out) some framing-specific texts. For the gain frame condition, this was “You started this round with 0 points and have gained ### points.” For the loss frame condition, it was “You started this round with 620 points and have lost ### points.”

### Results

We calculated a linear mixed logit model with the decision to invest as the dependent variable. The probability of losing, framing, and their interaction were the predictors. Random effects were used for participants and trials. For detailed information on the model, see Table S1 and Figure S1. We found a significant main effect for the probability of losing, *z* = 24.87, *p* < .001, indicating that participants were generally more likely to invest if the probability of losing was low. Importantly, there was also a significant main effect of framing, *z* = 3.83, *p* < .001, indicating that participants were less likely to invest in the gain frame condition than in the loss frame condition. These main effects were qualified by a significant interaction of framing and the probability of losing, *z* = -3.67, *p* < .001. The difference between the gain and loss frame conditions was smaller for low probabilities of losing.

### Discussion

The negative relation between the probability of losing and the participants’ likelihood of investing shows that our participants understood the VIP-Task and tried to maximize their payout. They were more likely to invest in good cards than in bad ones. Still, the participants did reinvest in bad hands, which can be interpreted as an escalation of commitment. In line with a loss aversion perspective on EoC, we found that participants were less likely to invest when they were in the gain frame rather than the loss frame condition (see Figure S1). Also, the difference between framing conditions was smaller for very low probabilities of losing. As with the responsibility effects in Experiment 1, this is likely due to a ceiling effect – participants were very likely to continue when the chance of losing was minimal.

**Supplementary Table 3**

*Mixed linear logit models estimating the decision to bet in the pilot study*

| Variable | | *OR* | *B* | | *SE B* | *z* | *p* | |
| --- | --- | --- | --- | --- | --- | --- | --- | --- |
| Intercept | |  | v | | 0.20 | 24.52 | <.001 | |
| Probability of losing | |  | -5.38 | | 0.18 | -29.56 | <.001 | |
| Framing ^a^ | |  | 1.22 | | 0.29 | 4.10 | <.001 | |
| Probability of losing × Framing ^a^ | |  | -0.98 | | 0.28 | -3.48 | <.001 | |
| Random effects (*s*^2^) | Participant: 0.88 | | | Trial: <0.01 | | | |  |

*Note.* ^a^ gain = 0, loss = 1.

*
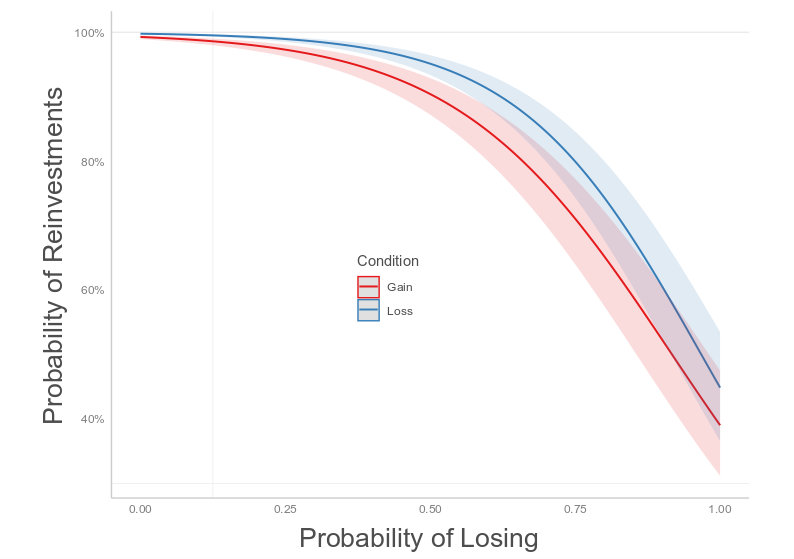
*

**Supplementary Figure 2**

*Reinvestments in the pilot study.* Note: Probability to reinvest as a function of the probability of losing and framing condition in Experiment 3, 95% confidence intervals are displayed.

# Experiment 3

## Predicting Expected Value

**Supplementary Table 4**

*Mixed linear model predicting the expected point value based on trial type, framing, and preferences in Study 3.*

| Predictors | B | CI | p |
| --- | --- | --- | --- |
| (Intercept) | 94.26 | 65.49 – 123.02 | <.001 |
| Trial type responsible ^a^ | 19.20 | 6.03 – 32.37 | .004 |
| Trial type optimal ^a^ | 78.98 | 66.76 – 91.19 | <.001 |
| Framing ^b^ | 1.22 | -10.56 – 13.00 | .840 |
| Preference ^c^ | 71.98 | 62.11 – 81.85 | <.001 |
| Numeracy | 2.99 | 0.42 – 5.57 | .023 |
| DOSPERT | 0.36 | -5.02 – 5.73 | .897 |
| Trial type responsible ^a^ × Framing ^b^ | 6.37 | -11.37 – 24.12 | .481 |
| Trial type optimal ^a^ × Framing ^b^ | 10.62 | -6.57 – 27.80 | .226 |
| Trial type responsible ^a^ × Preference ^c^ | -7.55 | -23.74 – 8.63 | .360 |
| Trial type optimal ^a^ × Preference ^c^ | -38.31 | -53.18 – -23.45 | <.001 |
| Framing ^b^ × Preference ^c^ | -2.27 | -16.36 – 11.83 | .753 |
| Trial type responsible ^a^ × Framing ^b^ × Preference ^c^ | -3.80 | -26.29 – 18.69 | .740 |
| Trial type optimal ^a^ × Framing ^b^ × Preference ^c^ | -12.19 | -33.43 – 9.05 | .261 |
| \| Random Effects (*s^2^*) \| Participant: 177.6 \| \| --- \| --- \|   *Note.* ^a^ The variables are dummy coded, random assignment trials are coded 0 on both variables; ^b^ gain = 0, loss = 1; ^c^ no-preference = 0, preference =1.” | | | |

## Conditional Effect of Responsibility

**Supplementary Table 5**

*Simple slope analyses in Study 3 conditional slopes and intercepts of Responsibility.*

|  |  |  | *B* | *SE B* | *z* | *p* |
| --- | --- | --- | --- | --- | --- | --- |
| Frame = gain | POL^a^ = .00 (- 1*SD*) | Cond. intercept | 2.58 | 0.14 | 18.02 | <.001 |
|  |  | Responsibility | 0.98 | 0.26 | 3.84 | <.001 |
|  | POL^a^ = .35 (*M*) | Cond. intercept | 1.64 | 0.13 | 13.03 | <.001 |
|  |  | Responsibility | 1.18 | 0.16 | 7.37 | <.001 |
|  | POL^a^ = .70 (*+ 1SD*) | Cond. intercept | 0.70 | 0.13 | 5.33 | <.001 |
|  |  | Responsibility | 1.38 | 0.14 | 9.80 | <.001 |
| Frame = loss | POL^a^ = .00 (- 1*SD*) | Cond. intercept | 3.29 | 0.17 | 19.85 | <.001 |
|  |  | Responsibility | 1.65 | 0.36 | 4.56 | <.001 |
|  | POL^a^ = .35 (*M*) | Cond. intercept | 2.21 | 0.14 | 16.22 | <.001 |
|  |  | Responsibility | 1.44 | 0.22 | 6.47 | <.001 |
|  | POL^b^ = .70 (*+ 1SD*) | Cond. intercept | 1.12 | 0.13 | 8.15 | <.001 |
|  |  | Responsibility | 1.21 | 0.15 | 8.49 | <.001 |

*Note.* ^a^ probability of losing.


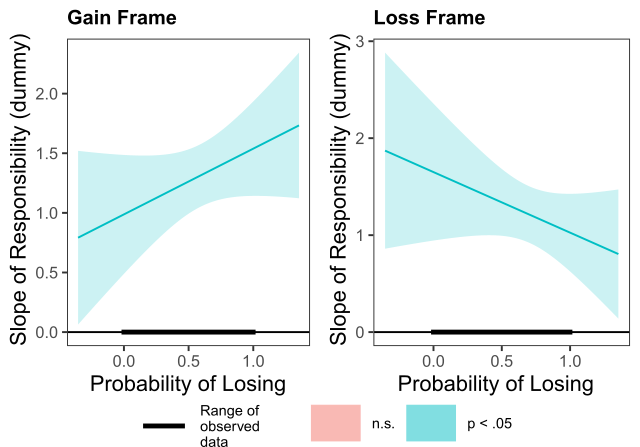
”

**Supplementary Figure 3**

*Johnson-Newman intervals for the conditional effect of responsibility on reinvestments in Experiment 2 with gain/loss framing and the probability of losing as the moderators.*
